# A second monoclinic modification of phenyl quinoxalin-2-yl ether

**DOI:** 10.1107/S1600536808031243

**Published:** 2008-10-22

**Authors:** Zanariah Abdullah, Seik Weng Ng

**Affiliations:** a Department of Chemistry, University of Malaya, 50603 Kuala Lumpur, Malaysia

## Abstract

The two aromatic systems in the title compound, C_14_H_10_N_2_O, enclose a dihedral angle of 77.9 (1)°, and the C—O—C inter-ring bond angle is 117.6 (1)°.

## Related literature

Another polymorph of this compound has recently been described in the *C*2/*c* space group; see Hassan *et al.* (2008 ▶).
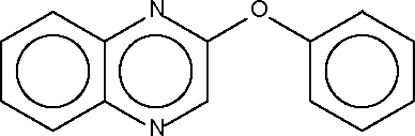


## Experimental

### 

#### Crystal data

C_14_H_10_N_2_O
*M* *_r_* = 222.24
Monoclinic, 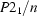

*a* = 7.9447 (2) Å
*b* = 6.5169 (1) Å
*c* = 20.2992 (5) Å
β = 91.983 (1)°
*V* = 1050.36 (4) Å^3^
*Z* = 4
Mo *K*α radiation
μ = 0.09 mm^−1^
*T* = 100 (2) K
0.40 × 0.20 × 0.10 mm

#### Data collection

Bruker SMART APEX diffractometer
Absorption correction: none
7016 measured reflections
2398 independent reflections
1960 reflections with *I* > 2σ(*I*)
*R*_int_ = 0.021

#### Refinement

*R*[*F* ^2^ > 2σ(*F* ^2^)] = 0.037
*wR*(*F* ^2^) = 0.114
*S* = 1.03
2398 reflections
154 parameters
H-atom parameters constrained
Δρ_max_ = 0.33 e Å^−3^
Δρ_min_ = −0.23 e Å^−3^

### 

Data collection: *APEX2* (Bruker, 2007 ▶); cell refinement: *SAINT* (Bruker, 2007 ▶); data reduction: *SAINT* ; program(s) used to solve structure: *SHELXS97* (Sheldrick, 2008 ▶); program(s) used to refine structure: *SHELXL97* (Sheldrick, 2008 ▶); molecular graphics: *X-SEED* (Barbour, 2001 ▶); software used to prepare material for publication: *publCIF* (Westrip, 2008 ▶).

## Supplementary Material

Crystal structure: contains datablocks global, I. DOI: 10.1107/S1600536808031243/im2084sup1.cif

Structure factors: contains datablocks I. DOI: 10.1107/S1600536808031243/im2084Isup2.hkl

Additional supplementary materials:  crystallographic information; 3D view; checkCIF report

## Figures and Tables

**Fig. 1.**
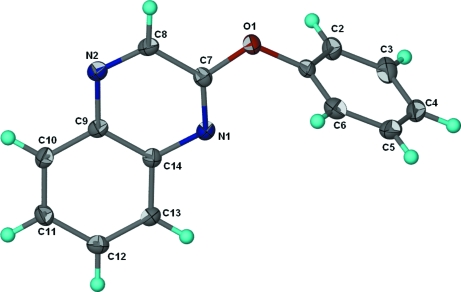
Thermal ellipsoid plot (Barbour, 2001) of C14H10N2O at the 70% probability level; hydrogen atoms are drawn as spheres of arbitrary radius.

## supplementary crystallographic information

### Comment

The compound was recently described in the *C*2/*c* space group with the two aromatic substituents in C_14_H_10_N_2_O enclosing a dihedral angle of 63.8 (1)°. The bond angle at oxygen measures to 118.2 (1)° (Hassan *et al.*, 2008). In the *P*2_1_/n modification described herein (Scheme I, Fig. 1), the two aromatic systems show a dihedral angle of 77.9 (1)° and they subtend an angle of 117.6 (1)° at oxygen.

### Experimental

The monoclinic modification was obtained when the *C*2/*c* modification of quinoxalinyl phenyl ether was recrystallized from ethanol in the presence of a small quantity of manganese acetate. Slow evaporation of the solvent gave colorless crystals mixed with unchanged manganese acetate.

### Refinement

Carbon-bound H-atoms were placed in calculated positions (C—H 0.95 Å) and were included in the refinement in the riding model approximation, with *U*(H) fixed at 1.2*U*(C).

### Crystal data

**Table:**

C_14_H_10_N_2_O	*F*(000) = 464
*M**_r_* = 222.24	*D*_x_ = 1.405 Mg m^−^^3^
Monoclinic, *P*2_1_/*n*	Mo *K*α radiation, λ = 0.71073 Å
Hall symbol: -P 2yn	Cell parameters from 2712 reflections
*a* = 7.9447 (2) Å	θ = 2.7–28.4°
*b* = 6.5169 (1) Å	µ = 0.09 mm^−^^1^
*c* = 20.2992 (5) Å	*T* = 100 K
β = 91.983 (1)°	Block, colorless
*V* = 1050.36 (4) Å^3^	0.40 × 0.20 × 0.10 mm
*Z* = 4	

### Data collection

**Table:**

Bruker SMART APEX diffractometer	1960 reflections with *I* > 2σ(*I*)
Radiation source: fine-focus sealed tube	*R*_int_ = 0.021
graphite	θ_max_ = 27.5°, θ_min_ = 2.0°
ω scans	*h* = −10→9
7016 measured reflections	*k* = −8→8
2398 independent reflections	*l* = −26→26

### Refinement

**Table:**

Refinement on *F*^2^	Primary atom site location: structure-invariant direct methods
Least-squares matrix: full	Secondary atom site location: difference Fourier map
*R*[*F*^2^ > 2σ(*F*^2^)] = 0.037	Hydrogen site location: inferred from neighbouring sites
*wR*(*F*^2^) = 0.114	H-atom parameters constrained
*S* = 1.03	*w* = 1/[σ^2^(*F*_o_^2^) + (0.0648*P*)^2^ + 0.2602*P*] where *P* = (*F*_o_^2^ + 2*F*_c_^2^)/3
2398 reflections	(Δ/σ)_max_ = 0.001
154 parameters	Δρ_max_ = 0.33 e Å^−^^3^
0 restraints	Δρ_min_ = −0.23 e Å^−^^3^

### Fractional atomic coordinates and isotropic or equivalent isotropic displacement parameters (Å^2^)

**Table:**

	*x*	*y*	*z*	*U*_iso_*/*U*_eq_	
O1	0.34884 (10)	0.24973 (13)	0.66577 (4)	0.0189 (2)	
N1	0.58503 (12)	0.26591 (14)	0.60174 (5)	0.0150 (2)	
N2	0.35723 (12)	0.23610 (14)	0.49088 (5)	0.0153 (2)	
C1	0.45193 (13)	0.28653 (19)	0.72247 (5)	0.0170 (3)	
C2	0.44960 (15)	0.48057 (19)	0.74939 (6)	0.0199 (3)	
H2	0.3836	0.5863	0.7292	0.024*	
C3	0.54551 (15)	0.5187 (2)	0.80653 (6)	0.0233 (3)	
H3	0.5461	0.6519	0.8255	0.028*	
C4	0.64037 (15)	0.3635 (2)	0.83596 (6)	0.0232 (3)	
H4	0.7066	0.3906	0.8749	0.028*	
C5	0.63876 (15)	0.1689 (2)	0.80870 (6)	0.0242 (3)	
H5	0.7029	0.0622	0.8293	0.029*	
C6	0.54360 (15)	0.1286 (2)	0.75122 (6)	0.0219 (3)	
H6	0.5419	−0.0047	0.7323	0.026*	
C7	0.42352 (15)	0.25028 (16)	0.60650 (5)	0.0148 (2)	
C8	0.30741 (14)	0.23391 (17)	0.55103 (6)	0.0158 (3)	
H8	0.1905	0.2211	0.5586	0.019*	
C9	0.52855 (14)	0.25143 (16)	0.48299 (5)	0.0139 (2)	
C10	0.59172 (15)	0.25296 (17)	0.41912 (5)	0.0160 (3)	
H10	0.5161	0.2467	0.3819	0.019*	
C11	0.76232 (15)	0.26345 (17)	0.41025 (6)	0.0174 (3)	
H11	0.8043	0.2630	0.3670	0.021*	
C12	0.87492 (15)	0.27483 (18)	0.46528 (6)	0.0179 (3)	
H12	0.9927	0.2808	0.4589	0.021*	
C13	0.81557 (14)	0.27741 (18)	0.52800 (6)	0.0167 (3)	
H13	0.8925	0.2878	0.5647	0.020*	
C14	0.64163 (14)	0.26477 (16)	0.53833 (5)	0.0141 (2)	

### Atomic displacement parameters (Å^2^)

**Table:**

	*U*^11^	*U*^22^	*U*^33^	*U*^12^	*U*^13^	*U*^23^
O1	0.0148 (4)	0.0292 (5)	0.0127 (4)	−0.0019 (3)	0.0008 (3)	−0.0024 (3)
N1	0.0146 (5)	0.0160 (5)	0.0144 (5)	0.0003 (3)	−0.0008 (4)	−0.0002 (3)
N2	0.0155 (5)	0.0137 (5)	0.0166 (5)	0.0004 (3)	−0.0011 (4)	−0.0005 (4)
C1	0.0119 (5)	0.0277 (6)	0.0116 (5)	−0.0025 (4)	0.0025 (4)	0.0000 (4)
C2	0.0208 (6)	0.0237 (6)	0.0153 (5)	−0.0008 (5)	0.0014 (4)	0.0026 (4)
C3	0.0253 (6)	0.0276 (7)	0.0170 (6)	−0.0061 (5)	0.0017 (5)	−0.0019 (5)
C4	0.0160 (6)	0.0396 (8)	0.0140 (5)	−0.0056 (5)	0.0002 (4)	0.0014 (5)
C5	0.0164 (6)	0.0368 (7)	0.0195 (6)	0.0047 (5)	0.0025 (4)	0.0076 (5)
C6	0.0199 (6)	0.0262 (7)	0.0199 (6)	0.0018 (5)	0.0042 (4)	−0.0001 (5)
C7	0.0168 (5)	0.0137 (5)	0.0139 (5)	0.0000 (4)	0.0014 (4)	−0.0007 (4)
C8	0.0134 (5)	0.0162 (6)	0.0177 (6)	0.0003 (4)	−0.0005 (4)	−0.0009 (4)
C9	0.0146 (5)	0.0113 (5)	0.0158 (6)	0.0011 (4)	−0.0008 (4)	0.0001 (4)
C10	0.0188 (6)	0.0148 (6)	0.0142 (5)	0.0015 (4)	−0.0024 (4)	0.0002 (4)
C11	0.0200 (6)	0.0186 (6)	0.0138 (5)	0.0017 (4)	0.0030 (4)	0.0013 (4)
C12	0.0147 (5)	0.0195 (6)	0.0196 (6)	0.0009 (4)	0.0023 (4)	0.0011 (4)
C13	0.0145 (5)	0.0192 (6)	0.0161 (6)	0.0009 (4)	−0.0020 (4)	0.0007 (4)
C14	0.0158 (6)	0.0121 (5)	0.0142 (5)	0.0007 (4)	−0.0002 (4)	0.0005 (4)

### Geometric parameters (Å, °)

**Table:**

O1—C7	1.3598 (14)	C5—H5	0.9500
O1—C1	1.4099 (13)	C6—H6	0.9500
N1—C7	1.2941 (15)	C7—C8	1.4346 (15)
N1—C14	1.3781 (14)	C8—H8	0.9500
N2—C8	1.2966 (15)	C9—C10	1.4066 (15)
N2—C9	1.3796 (15)	C9—C14	1.4165 (16)
C1—C2	1.3780 (17)	C10—C11	1.3751 (16)
C1—C6	1.3786 (17)	C10—H10	0.9500
C2—C3	1.3880 (16)	C11—C12	1.4086 (16)
C2—H2	0.9500	C11—H11	0.9500
C3—C4	1.3847 (18)	C12—C13	1.3730 (15)
C3—H3	0.9500	C12—H12	0.9500
C4—C5	1.383 (2)	C13—C14	1.4073 (16)
C4—H4	0.9500	C13—H13	0.9500
C5—C6	1.3931 (17)		
			
C7—O1—C1	117.58 (9)	O1—C7—C8	113.94 (10)
C7—N1—C14	115.20 (10)	N2—C8—C7	121.96 (11)
C8—N2—C9	116.40 (10)	N2—C8—H8	119.0
C2—C1—C6	122.08 (11)	C7—C8—H8	119.0
C2—C1—O1	117.68 (10)	N2—C9—C10	119.51 (10)
C6—C1—O1	120.14 (11)	N2—C9—C14	120.90 (10)
C1—C2—C3	118.77 (11)	C10—C9—C14	119.59 (10)
C1—C2—H2	120.6	C11—C10—C9	120.38 (10)
C3—C2—H2	120.6	C11—C10—H10	119.8
C4—C3—C2	120.30 (12)	C9—C10—H10	119.8
C4—C3—H3	119.8	C10—C11—C12	120.04 (11)
C2—C3—H3	119.8	C10—C11—H11	120.0
C5—C4—C3	119.98 (11)	C12—C11—H11	120.0
C5—C4—H4	120.0	C13—C12—C11	120.46 (11)
C3—C4—H4	120.0	C13—C12—H12	119.8
C4—C5—C6	120.32 (12)	C11—C12—H12	119.8
C4—C5—H5	119.8	C12—C13—C14	120.53 (10)
C6—C5—H5	119.8	C12—C13—H13	119.7
C1—C6—C5	118.53 (12)	C14—C13—H13	119.7
C1—C6—H6	120.7	N1—C14—C13	119.52 (10)
C5—C6—H6	120.7	N1—C14—C9	121.50 (10)
N1—C7—O1	122.03 (10)	C13—C14—C9	118.98 (11)
N1—C7—C8	124.02 (11)		
			
C7—O1—C1—C2	100.70 (12)	O1—C7—C8—N2	178.65 (10)
C7—O1—C1—C6	−82.79 (13)	C8—N2—C9—C10	179.47 (10)
C6—C1—C2—C3	1.36 (17)	C8—N2—C9—C14	−0.26 (15)
O1—C1—C2—C3	177.79 (10)	N2—C9—C10—C11	−178.53 (10)
C1—C2—C3—C4	−0.52 (17)	C14—C9—C10—C11	1.21 (16)
C2—C3—C4—C5	−0.52 (18)	C9—C10—C11—C12	−0.64 (16)
C3—C4—C5—C6	0.77 (18)	C10—C11—C12—C13	−0.59 (17)
C2—C1—C6—C5	−1.11 (17)	C11—C12—C13—C14	1.22 (17)
O1—C1—C6—C5	−177.45 (10)	C7—N1—C14—C13	−178.90 (10)
C4—C5—C6—C1	0.02 (17)	C7—N1—C14—C9	1.19 (15)
C14—N1—C7—O1	−179.82 (10)	C12—C13—C14—N1	179.46 (10)
C14—N1—C7—C8	−0.37 (15)	C12—C13—C14—C9	−0.63 (16)
C1—O1—C7—N1	5.91 (15)	N2—C9—C14—N1	−0.94 (16)
C1—O1—C7—C8	−173.59 (10)	C10—C9—C14—N1	179.33 (10)
C9—N2—C8—C7	1.10 (15)	N2—C9—C14—C13	179.15 (10)
N1—C7—C8—N2	−0.84 (17)	C10—C9—C14—C13	−0.58 (15)
